# Ventilator Settings and Monitoring Variables Associated with Extubation Failure in Critically Ill Patients: A Retrospective Cohort Study

**DOI:** 10.62675/2965-2774.20260327

**Published:** 2026-04-10

**Authors:** Emilio Steinberg, Luis Ignacio Garegnani, Damián Steinberg, Emilia Maniás, Pablo Lovazzano, Luca Mazzini, Jesica Nieto, Nahuel Lavítola, María Lucía Giménez, Mariano Setten

**Affiliations:** 1 Hospital Italiano Buenos Aires Argentina Hospital Italiano - Buenos Aires, Argentina.; 2 Sanatorio Anchorena San Martín Buenos Aires Argentina Sanatorio Anchorena San Martín - Buenos Aires, Argentina.; 3 Hospital Universitario CEMIC Sede Saavedra Buenos Aires Argentina Hospital Universitario CEMIC, Sede Saavedra - Buenos Aires, Argentina.; 4 Hospital Universitario CEMIC Sede Pombo de Rodriguez Buenos Aires Argentina Hospital Universitario CEMIC, Sede Pombo de Rodriguez - Buenos Aires, Argentina.; 5 Sanatorio de la Trinidad Mitre Buenos Aires Argentina Sanatorio de la Trinidad Mitre - Buenos Aires, Argentina

**Keywords:** Respiration, artificial, Weaning, Airway extubation, Respiratory insufficiency, Ventilators, mechanical

## Abstract

**Objective:**

Mechanical ventilation is an essential tool in the management of acute respiratory failure. Extubation is a critical event in the clinical course of patients, yet it may fail even in those who successfully complete a spontaneous breathing trial. This study aimed to evaluate which ventilator settings and monitoring variables are associated with extubation failure.

**Methods:**

This was a retrospective, multicenter cohort study, including patients from July 2021 to December 2022 across five intensive care units in Argentina. Adult patients (≥ 18 years old) who received at least 48 hours of mechanical ventilation and underwent an extubation were included. A multivariable logistic regression was performed with extubation failure - defined as reintubation or death within 7 days post-extubation– as the outcome variable. The analysis was adjusted for APACHE II score, type of spontaneous breathing trial, use of non-invasive ventilatory support after extubation, and COVID-19 as a reason for mechanical ventilation.

**Results:**

A total of 516 patients were analyzed, with an extubation failure rate of 35.8%. Oxygenation, measured by the oxygen saturation/fraction of inspired oxygen ratio prior to extubation was associated with a protective effect (OR 0.99, 95%CI 0.993 - 0.997) while higher positive end-expiratory pressure, fraction of inspired oxygen, and peak pressure showed a significant association with extubation failure (OR 1.26, 95%CI 1.09 - 1.45; OR 1.04, 95%CI 1.01 - 1.07; OR 1.04, 95%CI 1,003 - 1.07 respectively). Additionally, patients who failed had a higher incidence of *delirium* on the day of extubation and greater use of neuromuscular blocking agents during their intensive care unit stay.

**Conclusion:**

In critically ill patients undergoing mechanical ventilation, ventilator settings, monitoring parameters, and oxygenation status at the time of extubation may influence the likelihood of success. Better oxygenation appears protective, whereas elevated positive end-expiratory pressure and fraction of inspired oxygen may indicate increased vulnerability to extubation failure.

## INTRODUCTION

Mechanical ventilation (MV) is a fundamental tool in the management of acute respiratory failure. The most recent epidemiological study reported that approximately 43% of patients admitted to intensive care units (ICUs) require MV.^(1)^ The duration of MV is a critical factor, as both unnecessary prolongation and premature discontinuation are associated with adverse outcomes such as ventilator-associated pneumonia, other healthcare-associated infections, and ICU mortality.^(2-5)^

Weaning refers to the set of processes and evaluations aimed at discontinuing MV. This phase includes several stages, including the assessment of readiness to wean, the spontaneous breathing trial (SBT), and the removal of the artificial airway (extubation).^(6)^

Among patients undergoing weaning, a subgroup completes SBT and is extubated but still experiences extubation failure.^(2,6-8)^ The incidence of extubation failure varies between approximately 10% - 20%.^(7)^ These patients can be classified into those who fail due to upper airway obstruction ("airway failure") and those who develop recurrent acute respiratory failure.^(8)^

Numerous studies have identified risk factors associated with extubation failure.^(2,6-8)^ In 2023, the WEAN SAFE study reported novel findings on weaning failure. Specifically, ventilator settings and monitoring variables – such as respiratory rate, positive end-expiratory pressure (PEEP), and dynamic driving pressure (ΔP) - on the day of the SBT were associated with extubation failure.^(9)^

Mechanical ventilation provides critical support to ensure adequate gas exchange and to help reestablish lung volume.^(10)^ However, its use is not free from complications. To minimize the impact of MV on respiratory muscle function, current recommendations include strategies for "diaphragm-protective ventilation", which aim to maintain an appropriate respiratory load while preserving lung-protective settings,^(11)^ with ongoing consideration of the weaning process.^(5,6,10)^

Ventilator settings and monitoring, therefore, play a central role in achieving these goals. Relying on variables with low sensitivity and specificity to detect over-assistance may lead to underestimating the actual respiratory effort.^(12)^ Moreover, patients with more severe illness or suboptimal conditions for weaning (e.g., impaired respiratory mechanics, gas exchange, or metabolic status) may still fulfill criteria for initiating an SBT, yet require higher levels of support (PEEP, inspiratory pressure, fraction of inspired oxygen [FiO_2_]) to maintain respiratory function.^(5,9)^

In summary, even when readiness criteria for weaning are met, the strategy used for ventilator programming and monitoring may be associated with extubation failure. Within this context, we conducted a retrospective cohort study with the primary objective of to evaluating which ventilator settings and monitoring variables are associated with extubation orotracheal failure.

## METHODS

We conducted a retrospective, multicenter cohort study (NCT06683781) between July 1, 2021, and December 31, 2022. The study methods adhered to the Strengthening the Reporting of Observational Studies in Epidemiology (STROBE) guidelines.^(13)^

### Ethics Statement

The Ethics Committees of all participating institutions approved the study and waived informed consent requirements due to its retrospective design. The study was conducted in accordance with the Declaration of Helsinki (2008) and its subsequent amendments.

### Sample

Five centers participated in the study: four located in the City of Buenos Aires and one in the Province of Buenos Aires, Argentina. We included adult patients (≥ 18 years) admitted to the ICU, who had received at least 48 hours of MV and underwent an extubation, regardless of the outcome. Patients with missing data regarding exposure and/or outcome variables were excluded.

### Procedure

For this study, we specifically designed a database using the REDCap platform. Data was collected from electronic medical records, including demographic information and patient characteristics at ICU admission, as well as ventilator settings and monitoring variables. These were recorded both at the initiation of MV (ventilatory mode, tidal volume, inspiratory pressure, PEEP, and set respiratory rate; exhaled tidal volume, peak and plateau pressures, static driving pressure, dynamic driving pressure, SpO_2_ and FiO_2_), and immediately prior to extubation (the same variables, except for static measurements such as plateau pressure and ΔP). Patients were followed until ICU discharge or death.

### Definitions and Outcomes

#### Outcome, Exposure, and Confounding Variables

The primary outcome was extubation failure, defined as the need for reintubation or death within 7 days after extubation.^(4)^ The reasons for failure were categorized as either "airway failure" or "respiratory failure".^(8)^ Exposure variables encompassed ventilator settings and MV monitoring parameters documented in the period preceding extubation. For this study, "preceding" was operationally defined as the most recent entry in the patient's medical chart.

#### Statistical Analysis

Continuous variables were reported as mean and standard deviation or median and interquartile range, depending on their distribution. Normality was assessed by visual inspection of histograms and standard probability plots, and by the Shapiro-Wilk test. Categorical variables were reported as absolute and relative frequencies. Continuous variables were compared using the Student's *t*-test or the Mann-Whitney U test, depending on their distribution. Categorical variables were compared using the Chi-squared test or Fisher's exact test, as appropriate. Following an association model, we performed univariable logistic regression to evaluate the association between ventilator settings and monitoring variables with the primary outcome (extubation failure). In addition, we conducted a multivariable logistic regression analysis, adjusting for severity calculated by Acute Physiology and Chronic Health Evaluation (APACHE) II, type of SBT, the use of non-invasive respiratory support post-extubation, and coronavirus disease 2019 (COVID-19) as a reason for initiation of MV, all causes considered as potential confounders when discussing extubation failure.^(4,14-17)^ The strength of association was expressed as an odds ratio (OR) with a 95% confidence interval (95%CI). All statistical tests were two-tailed, with p < 0.05 as the criterion for statistical significance. Statistical analyses were performed using STATA version 14 (StataCorp®, LLC, USA).

## RESULTS

During the study period, 516 participants met the eligibility criteria and underwent a first extubation; of these, 331 were successful, and 185 failed, yielding an extubation failure rate of 35.8%. Of those 185 patients, 169 were re-intubated, and 16 died within the first 7 days. Clinical characteristics at ICU admission are presented in table 1. Initial ventilator settings and monitoring variables are detailed in table 1S (Supplementary Material). Although the univariate analysis did not demonstrate statistically significant differences in the reasons for initiating MV, patients with COVID-19 showed a consistent trend toward higher rates of extubation failure (p = 0.057; Table 2S [Supplementary Material]). Table 2 presents the results of the logistic regression models, including both univariable and multivariable analyses. Higher PEEP and FiO_2_ levels prior to extubation were significantly associated with failure: for each additional cmH_2_O of PEEP, the odds of failure increased by 26%, and for each 1% increase in FiO_2_, the odds increased by 4%. In contrast, both the oxygen saturation (SpO_2_)/FiO_2_ and SpO_2_ demonstrated a protective effect. For every one-unit increase in the SpO_2_/FiO_2_, the odds of failure decreased by 1%, and for each additional percentage point in oxygen saturation, the odds of failure decreased by 19%.

**Table 1:** Clinical characteristics of patients at intensive care unit admission

	Population (n = 516)	Extubation success (n = 331)	Extubation failure (n = 185)	p value
Age (years)	67.3 [52.4 - 77.1]	63.5 [47.9 - 75.6]	72.6 [60 - 79.48]	< 0.001
Sex, female	253 (49.03)	159 (48.03)	94(50.8)	0.540
Reason for initiation of MV				
On chronic respiratory failure	21 (4.07)	15 (4.53)	6 (3.24)	0.690
*De novo* acute respiratory failure	406 (78.68)	258 (77.95)	148 (80)	
Acute neurological disease	81 (15.7)	54 (16.31)	27 (14.59)	
Neuromuscular disease	8 (1.55)	4 (1.21)	4 (2.16)	
APACHE II	16 [11 - 22]	16 [10 - 22]	16 [12 - 23]	0.160
Charlson	4 [1 - 5]	3 [1 - 5]	4 [2 - 6]	< 0.05
BMI (kg/m_2_)	26.5 [22.8 - 30.7]	26.15 [22.8 - 29.7]	27.07 [22.8 - 30.6]	0.390

MV- mechanical ventilation; APACHE II - Physiology and Chronic Health Evaluation II; BMI - body mass index. Results expressed as median [interquartile range] or n (%).

**Table 2:** Univariable logistic regression of ventilator settings and monitoring variables associated with extubation failure, and adjusted for type of spontaneous breathing trial, post-extubation non-invasive respiratory support, COVID-19 as a cause of respiratory failure, and APACHE II

Variable	OR	95%CI	p value	Adjusted OR	Adjusted 95%CI	Adjusted p value
Ventilatory mode	0.85	0.69 - 1.06	0.17	0.8	0.63 - 1	0.06
Inspiratory pressure	0.97	0.92 - 1.03	0.49	0.98	0.92 - 1.04	0.58
PEEP	1.18	1.04 - 1.35	0.01	1.25	1.08 - 1.44	< 0.05
Peak pressure	1.03	0.99 - 1.06	0.06	1.03	1.002 - 1.07	< 0.05
Set respiratory rate	1.07	0.96 - 1.18	0.17	1.03	0.93 - 1.16	0.51
Monitored respiratory rate	1.03	0.98 - 1.07	0.14	1.02	0.98 - 1.06	0.25
Exhaled V_T_	0.99	0.99 - 1	0.4	0.99	0.99 - 1	0.4
ΔP_DYN_	1.02	0.98 - 1.06	0.18	1.02	0.99 - 1.06	0.14
SpO_2_	0.8	0.72 - 0.88	< 0.001	0.81	0.73 - 0.9	< 0.001
FiO_2_	1.05	1.02 - 1.08	< 0.001	1.04	1.01 - 1.08	< 0.05
SpO_2_/FiO_2_	0.99	0.993 - 0.997	< 0.001	0.99	0.993 - 0.998	< 0.05

OR - odds ratio; 95%CI - 95% confidence Interval; PEEP - positive end-expiratory pressure; V_T_ - tidal volume; ΔP_DYN_ - dynamic driving pressure; SpO_2_ - partial oxygen saturation; FiO_2_ - fraction of inspired oxygen; SpO_2_/FiO2 - partial oxygen saturation to fraction of inspired oxygen ratio.

Table 3 summarizes additional findings and clinical outcomes. We observed statistically significant differences between patients who failed extubation and those who did not, in terms of delirium on the day of extubation, use of neuromuscular blocking agents during ICU stay, days to extubation, duration of MV, and ICU mortality.

**Table 3:** Clinical findings and outcomes

	Population (n = 516)	Extubation success (n = 331)	Extubation failure (n = 185)	p value
CAM-ICU the day of 1IS				
Positive	219 (42.52)	124 (37.58)	95 (51.35)	0.007
Negative	270 (52.43)	190 (57.87)	80 (43.24)	
Not valuable	26 (5.05)	16 (4.85)	10 (5.41)	
Use of NMB	90 (17.51)	44 (13.37)	46 (24.86)	< 0.001
Days until 1IS	5 [3 - 8]	5 [3 - 8]	6 [3 - 9]	0.430
Days until extubation	6 [4 - 9]	5 [4 - 8]	7 [4 - 10]	< 0.05
ICU LOS	17 [10 - 29]	14 [10 - 22]	26 [15 - 42]	< 0.001
Mortality	102 (19.84)	21 (6.38)	81 (43.78)	< 0.001

CAM-ICU - Confusion Assessment Method for the Intensive Care Unit; 1IS - first separate attempt; NMB - neuromuscular blockade; ICU LOS - intensive care unit length of stay. Results expressed as n (%) or median [interquartile range].

## DISCUSSION

This study reports the association between ventilator settings and monitoring variables immediately prior to extubation and extubation failure, adjusting for APACHE II, type of SBT, the use of non-invasive support post-extubation, and COVID-19 as a reason for initiation of MV, in a general population of critically ill patients. Our main findings can be summarized as follows: better oxygenation prior to extubation had a protective effect; higher PEEP and FiO_2_ levels were associated with extubation failure; patients who failed extubation had higher rates of delirium on the day of extubation, more frequent use of neuromuscular blockers, longer time to extubation, more days on MV, and increased mortality.

The reported rate of extubation failure in general ICU populations ranges from 10% to 20% in the literature, both worldwide^(7)^ and in Argentina.^(18)^ In our study, the failure rate was notably higher, reaching approximately 35%. A possible explanation for this finding is the inclusion of patients with COVID-19. In our cohort, 15% of patients required MV for COVID-19. Extubation failure in this subgroup exceeded 40%, which is notably higher than the ∼30% reported in national^(17)^ and international series.^(19)^ Failure rates among other groups, such as post-operative and septic patients, were also remarkable. These outcomes may be partly explained by the study period, as some pandemic-related changes in clinical practice and the considerable strain on the healthcare system could have influenced the results.^(20)^

Patients requiring MV often exhibit impaired gas exchange, particularly oxygenation, which is reflected by decreased arterial partial pressure of oxygen (PaO_2_) and SpO_2_ levels. These alterations are well-known and directly linked to the underlying pathophysiology.^(21)^ Oxygenation influences respiratory drive, and its impairment can lead to increased work of breathing.^(22)^ Critically ill patients frequently develop general deconditioning, including peripheral and respiratory muscle weakness.^(23,24)^ Although we did not measure muscle strength or respiratory mechanics, this combination of factors may help explain the association between oxygenation and extubation failure. Our findings align with those of the WEAN SAFE study, which was the first to report that patients with better oxygenation had lower extubation failure rates.^(9)^ However, unlike our study, WEAN SAFE used the PaO_2_/FIO_2_. While this index is widely used in clinical practice and the literature, it has limitations. Its value is influenced by both F_IO2_ and cardiac output, particularly in patients with acute respiratory distress syndrome (ARDS), where its use has been standardized.^(25,26)^ Additionally, it requires arterial blood sampling, which is invasive, painful, and costly—resources not always universally available.^(27)^ In this context, the SpO_2_/FiO_2_ has been proposed as an alternative. Although it also has limitations^(28,29)^ and still requires further validation, it offers a non-invasive, real-time tool for assessing oxygenation.^(30,31)^ Ventilator settings can be divided into those that support ventilation (e.g., tidal volume, inspiratory pressure, respiratory rate) and those that affect oxygenation (PEEP and FiO_2_). Therefore, it is unsurprising that patients with poorer oxygenation prior to extubation were managed with higher PEEP and FiO_2_ levels. Moreover, the withdrawal of positive pressure itself may lead to lung collapse and deterioration of gas exchange.^(32)^

The effect of PEEP on diaphragmatic function largely depends on its pulmonary impact: if it restores lung volume, diaphragm apposition improves, enhancing its mechanical efficiency. If not, the diaphragm flattens, reducing its zone of apposition and mechanical advantage, thereby impairing its pressure-generating capacity.^(33)^ In this context, it has been proposed that patients weaned from unnecessarily high levels of PEEP may be at risk of ventilator-induced diaphragm dysfunction: PEEP may induce longitudinal atrophy,^(34)^ and its abrupt removal during SBT or extubation may cause overstretching of weakened fibers, resulting in muscle dysfunction. This mechanism has been proposed as a possible contributor to weaning failure linked to diaphragmatic weakness.^(33)^ From another perspective, patients with greater respiratory compromise might require higher levels of PEEP and FiO_2_. Under these circumstances, there is an increased risk of ventilator-associated lung and diaphragmatic injury, which could ultimately affect the success of ventilatory support withdrawal. These findings challenge the WEAN SAFE-proposed weaning eligibility criteria.^(9)^ While the 2001^(5)^ and 2007^(6)^ guidelines recommend a PEEP ≤ 8cmH_2_O for weaning readiness, WEAN SAFE raises this threshold to 10cmH_2_O. This may overestimate the patient's ability to breathe spontaneously. Our findings are consistent with previous studies and support authors who advocate for a more cautious interpretation of this threshold.^(35)^

Delirium is common among ICU patients, with a prevalence of around 30%,^(36)^ increasing significantly in those requiring MV.^(37)^ Its association with poor clinical outcomes is well established.^(38)^ We found only one study reporting an association between delirium prior to extubation and extubation failure; however, the authors used the Intensive Care Delirium Screening Checklist (ICDS).^(39,40)^ In our study, although we did not conduct a formal association analysis, we observed a statistically significant difference in the presence of *delirium* at the time of extubation between patients who failed and those who did not, as measured by the Confusion Assessment Method for the ICU (CAM-ICU).^(37)^ While PADIS guidelines do not specify which tool to use – ICDS or CAM-ICU – evidence suggests that CAM-ICU may be more accurate.^(41,42)^ Additionally, these guidelines recommend using validated tools, and CAM-ICU is the only instrument validated in Spanish.^(40,41,43,44)^ Our findings suggest that further research is needed to explore the potential role of *delirium* in extubation failure. The differences observed in the univariate analysis between patients who failed extubation and those who succeeded, regarding delirium and exposure to neuromuscular blocking agents during MV, are noteworthy. However, because these factors are consequences of patient management rather than parameters of ventilator settings or monitoring, they were not included in the multivariable regression model.

When analyzing the duration of MV, the absence of differences in the number of days until the first separation attempt contrasts with the differences observed in time to extubation between patients who failed and those who succeeded in removing the endotracheal tube. This pattern supports the hypothesis that failure of the initial attempt may set the stage for future extubation failure.^(45)^ Similar to our results, the WIND study reported a stepwise increase in reintubation rates, with 0% in Group 1, 25.6% in Group 2, and 63% in Group 3.^(4)^

This study has several limitations. First, it’ is a retrospective design. Second, the external validity could be affected by the small number of participating centers and their geographic location: all are located in a specific area of Argentina. Additionally, we did not require sedation or weaning protocols as inclusion criteria, which may have influenced the relationship between ventilator monitoring and extubation outcomes. The latter could be considered an important limitation. Lastly, variations in monitoring standards between centers may have affected data recording. To address these limitations, we implemented a comprehensive data quality control strategy, including: strict definitions and standardization of variables, training sessions with principal investigators from each center, validation of variables through programmed range and unit checks, and REDCap's built-in data quality controls supplemented by custom scripts developed by the study's principal investigator.

## CONCLUSION

In a general intensive care unit population requiring mechanical ventilation, oxygenation, as measured by the SpO_2_/FiO_2_, was associated with a protective effect against extubation failure. In contrast, higher positive end-expiratory pressure and FiO_2_ levels were associated with an increased risk of failure. Additionally, patients who failed extubation had a higher incidence of *delirium* on the day of extubation and greater use of neuromuscular blocking agents.

## Data Availability

The contents underlying the research text are included in the manuscript.
